# Supplementary figures and images for: Gene flow from Fraxinus cultivars into natural stands of Fraxinus pennsylvanica occurs range-wide, is regionally extensive, and is associated with a loss of allele richness
Source: PLoS One. 2024 May 16;19(5):e0294829. doi: 10.1371/journal.pone.0294829 (PMC11098341; doi:10.1371/journal.pone.0294829)

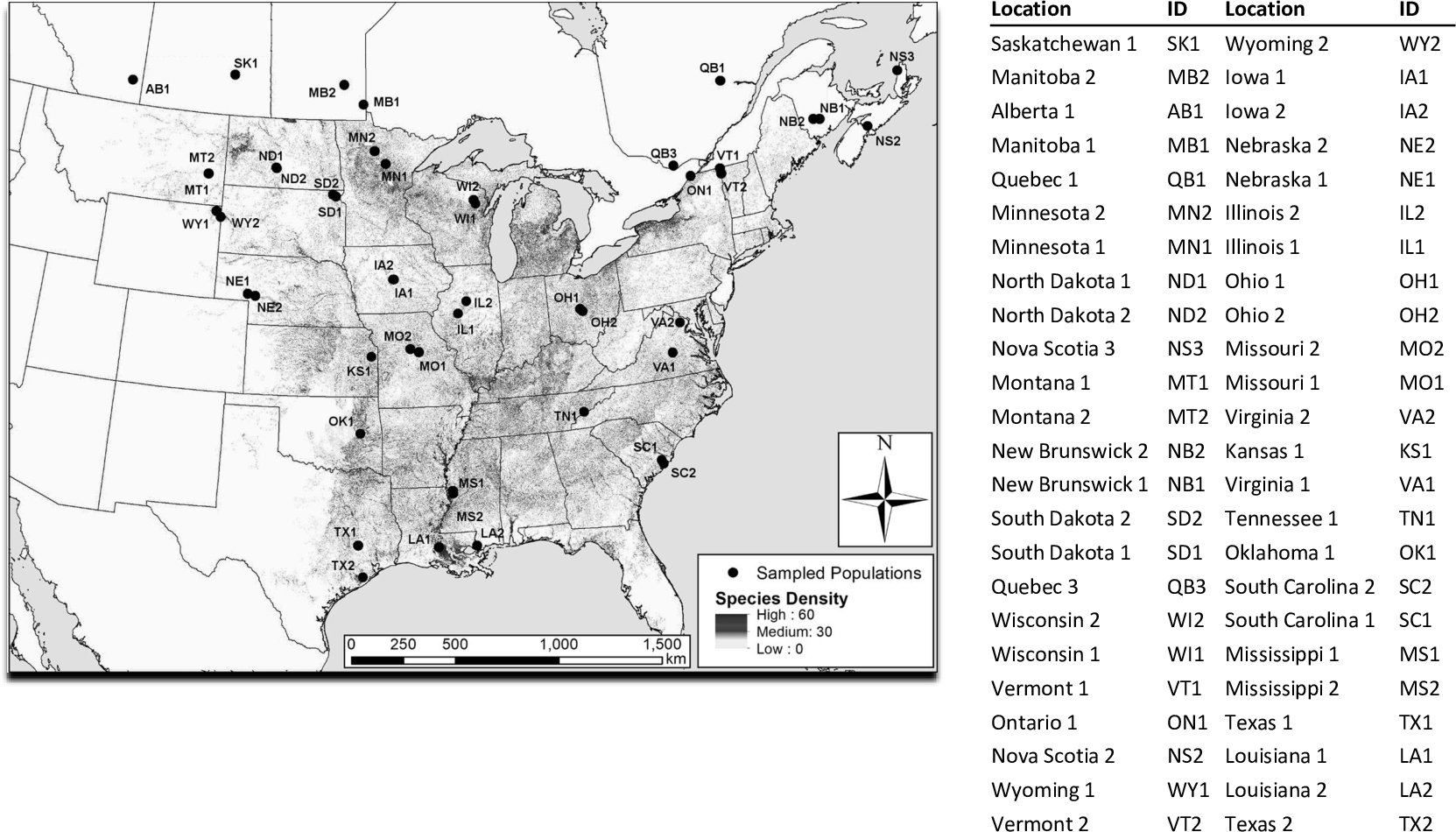

Supplement: S1 Fig — Thirty-eight site locations are labeled after the state in the United States in which they occur. Ten site locations, those in Alberta, Saskatchewan, Manitoba, Ontario, Quebec, New Brunswick, and Nova Scotia, are labeled after the Canadian provinces in which they occur. Species density data shown for the United States only. (TIF) [file pone.0294829.s001.tif]

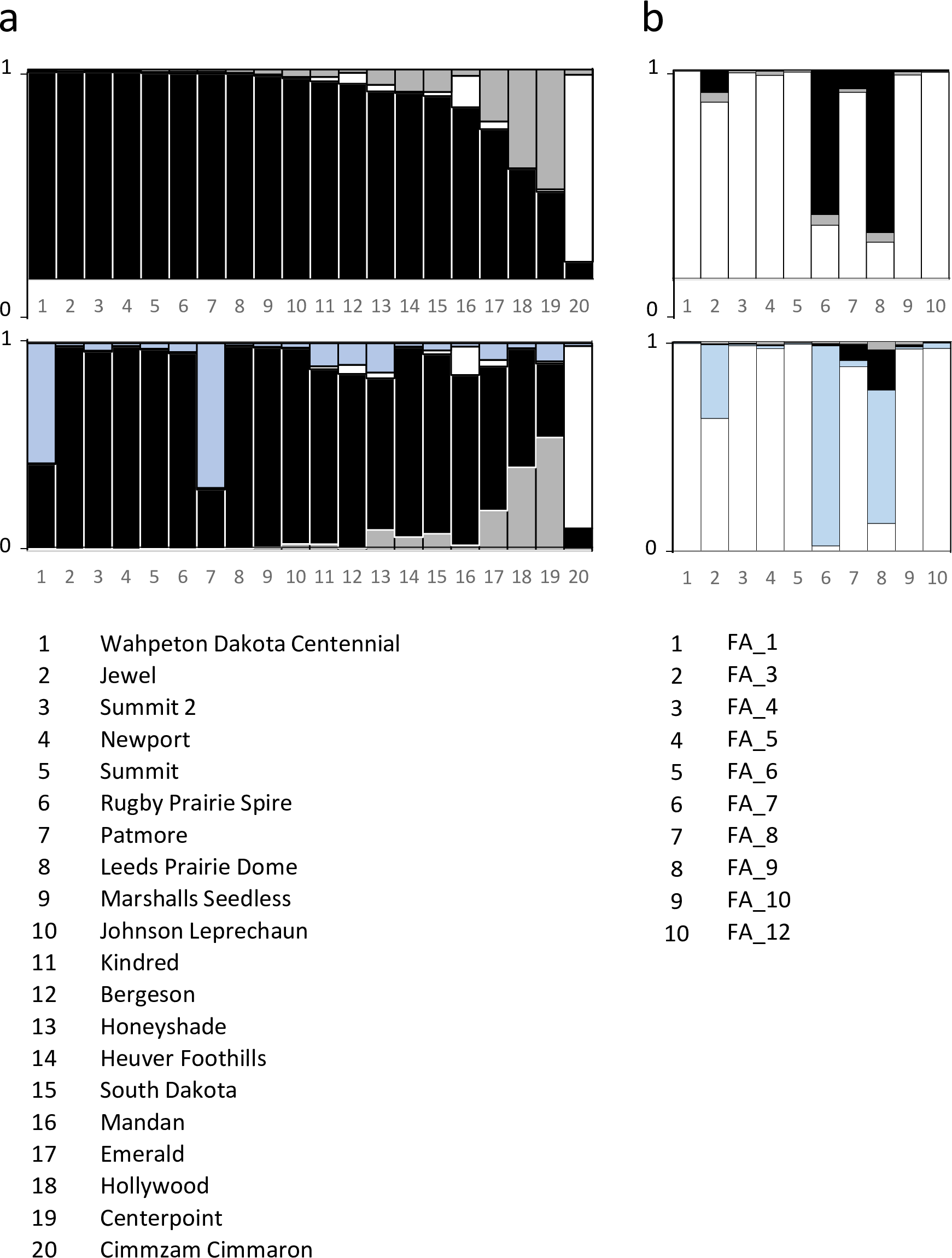

Supplement: S2 Fig — Panel a) Genetic groups and admixture inferred at K = 3. Panel b) Genetic groups and admixture at K = 4. (TIF) [file pone.0294829.s002.tif]
