# Supplementary material for: Gene flow from Fraxinus cultivars into natural stands of Fraxinus pennsylvanica occurs range-wide, is regionally extensive, and is associated with a loss of allele richness
Source: PLoS One. 2024 May 16;19(5):e0294829. doi: 10.1371/journal.pone.0294829 (PMC11098341; doi:10.1371/journal.pone.0294829)
Supplement: S1 Table — (DOCX) [file pone.0294829.s003.docx]

**S1 Table. Provenance and other descriptors for the cultivars included in this study**

| **Cultivar Name** | **ID** | **Original Source Location** | **Sex** | **Patent, Trademark or Publication** | **Source^a^** | **Accession^b^** |
| --- | --- | --- | --- | --- | --- | --- |
| Lednaw Aerial™ | - | Madison, Wisconsin | M | US PP7120_P | Schmidt, J. Frank & Son Co. | 19920133 |
| Bergeson | Brg | Fertile, Minnesota | M | US PP4904_P | Bailey Nurseries Inc. | 19920345 |
| Cimmzam Cimmaron® | CC | Perry, Ohio | M | US PP8077_P | Schmidt, J. Frank & Son Co. | 19940255 |
| Centerpoint | CP | Centerpoint, Iowa | M | Santamour and Mcardle 1983 | Schmidt, J. Frank & Son Co. | 19960459 |
| Emerald | Em | Arlington, Nebraska | M | Santamour and Mcardle 1983 | Schmidt, J. Frank & Son Co. | 19880133 |
| Heuver Foothills™ | HF | Montana | M | US PP13262 P2 | Bailey Nurseries Inc. | 19990571 |
| Honeyshade | HS | Arlington Heights, Illinois | M | US PP3385 P | Bailey Nurseries Inc. | 19750316 |
| Hollywood | HW | Cedar Rapids, Iowa | M | Santamour and Mcardle 1983 | Linn County Nurseries | 19710240 |
| Jewel | JWL | Lake City, Minnesota | F | Santamour and Mcardle 1983 | Jewell Nurseries Inc. | 19780276 |
| Johnson Leprechaun™ | JL | Menomonee Falls, Wisconsin | M | US PP9136_P | Schmidt, J. Frank & Son Co. | 19960476 |
| Kindred | Kin | Kindred, North Dakota | M | Santamour and Mcardle 1983 | Cross Nurseries Inc. | 19800257 |
| Leeds Prairie Dome™ | LPD | North Dakota | M | Trademark Registration Number 1838044 | Bailey Nurseries Inc. | 19990572 |
| Mandan | Man | North Dakota or Montana | F | USDA Green Ash Plant Guide | USDA Northern Great Plains field station, Mandan, ND | 19790544 |
| Marshalls Seedless | MS | Unknown | M | Gilman and Watson 1993 | Bailey Nurseries Inc. | 19750317 |
| Patmore | Pat | Vegreville, Alberta | M | US PP4684 P | Bailey Nurseries Inc. | 19830549 |
| Newport | New | St. Paul, Minnesota | M | Santamour and Mcardle 1983 | Bailey Nurseries Inc. | 19770200 |
| Rugby Prairie Spire® | RPS | North Dakota | M | Trademark Registration Number 1837444 | Bailey Nurseries Inc. | 19930379 |
| South Dakota | SD | Fertile, Minnesota | NR^c^ | Santamour and Mcardle 1983 | Bergeson Nursery | 19760174 |
| Summit | Sum | Stillwater, Minnesota | M | Santamour and Mcardle 1983 | Bailey Nurseries Inc. | 19750318 |
| Wahpeton Dakota Centennial™ | WDC | North Dakota | M | Trademark Registration Number 1853762 | Bailey Nurseries Inc. | 19930378 |

^a^Minnesota Landscape Arboretum source ^b^Minnesota Landscape Arboretum accession number ^c^Not reported
