# Supplementary material for: Gene flow from Fraxinus cultivars into natural stands of Fraxinus pennsylvanica occurs range-wide, is regionally extensive, and is associated with a loss of allele richness
Source: PLoS One. 2024 May 16;19(5):e0294829. doi: 10.1371/journal.pone.0294829 (PMC11098341; doi:10.1371/journal.pone.0294829)
Supplement: S2 Table — Linear regression model summary statistics using allele richness as the response variable and cultivar incidence as the predictor variable. (DOCX) [file pone.0294829.s004.docx]

| SUMMARY OUTPUT | | All 48 populations | | | |  | |  |
| --- | --- | --- | --- | --- | --- | --- | --- | --- |
|  |  |  | | |  | |  | |
| *Regression Statistics* | |  |  |  |  | |  | |
| Multiple R | 0.388703 |  |  |  |  | |  | |
| R Square | 0.15109 |  |  |  |  | |  | |
| Adjusted R Square | 0.132635 |  |  |  |  | |  | |
| Standard Error | 0.920543 |  |  |  |  | |  | |
| Observations | 48 |  |  |  |  | |  | |
|  |  |  |  |  |  | |  | |
| ANOVA |  |  |  |  |  | |  | |
|  | *df* | *SS* | *MS* | *F* | *Significance F* | |  | |
| Regression | 1 | 6.937766593 | 6.937767 | 8.18713 | 0.0063276 | |  | |
| Residual | 46 | 38.98035841 | 0.847399 |  |  | |  | |
| Total | 47 | 45.918125 |  |  |  | |  | |
|  |  |  |  |  |  | |  | |
|  | *Coefficients* | *Std Error* | *t Stat* | *P-value* | *Lower 95%* | | *Upper 95%* | |
| Intercept | 7.214009 | 0.182448318 | 39.54001 | 3.67E-37 | 6.84675941 | | 7.58125824 | |
| X Variable 1 | -2.703893 | 0.944982214 | -2.861316 | 0.006328 | -4.6060434 | | -0.80174232 | |
|  |  |  |  |  |  | |  | |
|  |  |  |  |  |  | |  | |
|  |  |  |  |  |  | |  | |
| SUMMARY OUTPUT | | Only the 33 populations with cultivar parentage | | | | |  | |
|  |  |  |  | |  | |  | |
| *Regression Statistics* | |  |  |  |  | |  | |
| Multiple R | 0.398633 |  |  |  |  | |  | |
| R Square | 0.158909 |  |  |  |  | |  | |
| Adjusted R Square | 0.131777 |  |  |  |  | |  | |
| Standard Error | 0.863474 |  |  |  |  | |  | |
| Observations | 33 |  |  |  |  | |  | |
|  |  |  |  |  |  | |  | |
| ANOVA |  |  |  |  |  | |  | |
|  | *df* | *SS* | *MS* | *F* | *Significance F* | |  | |
| Regression | 1 | 4.366807863 | 4.366808 | 5.856874 | 0.02157013 | |  | |
| Residual | 31 | 23.11319214 | 0.745587 |  |  | |  | |
| Total | 32 | 27.48 |  |  |  | |  | |
|  |  |  |  |  |  | |  | |
|  | *Coefficients* | *Std Error* | *t Stat* | *P-value* | *Lower 95%* | | *Upper 95%* | |
| Intercept | 7.234104 | 0.267020307 | 27.09196 | 3.81E-23 | 6.6895121 | | 7.77869511 | |
| X Variable 1 | -2.775219 | 1.14673855 | -2.420098 | 0.02157 | -5.11400818 | | -0.4364308 | |

Summary Statistics: Predictor variable: cultivar incidence, Response variable: allele richness

Plots of summary statistics

All 48 populations Only the 33 populations with cultivar gene flow
