# Supplementary material for: Gene flow from Fraxinus cultivars into natural stands of Fraxinus pennsylvanica occurs range-wide, is regionally extensive, and is associated with a loss of allele richness
Source: PLoS One. 2024 May 16;19(5):e0294829. doi: 10.1371/journal.pone.0294829 (PMC11098341; doi:10.1371/journal.pone.0294829)
Supplement: Attachment — Submitted filename: Response to Reviewers.docx [file pone.0294829.s006.docx]

Response to the reviewers

Reviewer #1: This manuscript needed to restructured and written again as its present form create lots of confusions among the reader.

We have restructured the abstract and the introduction to clarify what the investigation was about, what the research questions were, and the method we used to address these questions. In the revised abstract, in sentences 1 and 2 we write “Undetected cultivar gene flow, if extensive, could significantly lower genetic diversity within populations, suppress differentiation between populations, generate interspecific admixture not driven by long-standing natural processes, and affect the impact of abiotic and biotic threats. We used 16 EST-SSR markers to genotype 48 naturally regenerated populations of *F. pennsylvanica* distributed across the native range (1291 trees), 19 *F. pennsylvanica* cultivars, and one *F. americana* L. (white ash) cultivar to detect cultivar propagule dispersal into these populations. “

1. In introduction author needs to first explain the Green ash and white ash plant separately with their origin, ecology, uses and function in that region.

The investigation was not focused on the distinctions between *F. pennsylvanica* and *F. americana*, as is evident in the title, which we have not changed. We revised the abstract and the introduction to make this clear. Although a result of our investigation was the detection of admixture, this detection does not does not change our main result, the detection of evidence for cultivar gene flow into natural stands and the negative association of cultivar gene flow with allele richness. We included the white ash comparators because we were aware of the existing evidence for the difficulty of discerning the species by morphological and site criteria alone, as stated in the original text. Previous investigations focused on the difficulty of distinguishing these species are cited in the text. Our results pertinent to species admixture are not novel and are not presented as such. Our result does add complexity to the question of what constitutes the *F. pennsylvanica* gene pool, which we address in the discussion.

2. Authors have used the words green ash, white ash, and their scientific names in the whole manuscript carelessly which create confusion among the reader and have to go back in the introduction which one is green ash and which one is white ash. The name should be synchronized and one name should be used in all the manuscript.

The reviewer is correct. We have revised the text and now use the designations *F. pennsylvanica* and *F. americana* consistently throughout the manuscript.

3. The last paragraph of introduction line 94-102, author have included the lines of methods, results, and conclusion while he should justify why they have conducted such study and what was the hypothesis behind the study?

This comment and the reviewer’s first comment indicate that we did not clearly state what the scientific issue was (gene flow from cultivars in natural stands), and what the potential impact of the issue might be (loss of genetic variation). We have restructured the abstract and the introduction to clarify what the investigation was about, what the research questions were, and then state the method we used to address these questions.

4. Are cultivar and hybrid same thing? Than use only one terminology.

The words ‘cultivar’ and ‘hybrid’ are certainly not the same thing. We did not make this clear and carelessly used the word ‘hybrid’ in two places in the text. We have added text explaining what the word ‘cultivar’ means in the introduction and explain that the cultivar designation does not necessarily imply species purity. We deleted the two uses of the word ‘hybrid’ in the original submission. The word ‘hybrid’ no longer appears in the text. Individuals for which we have evidence of interspecific ancestry are now consistently labeled ‘admixed’.

5. In Method section author should clearly mentioned how many accession he had taken of green ash, suspected cultivar, and White ash from 48 population

This comment indicates that we did not make our methods as clear as we could have. In the revised methods section, we clearly define “population”, indicate that all the individuals in the defined population were genotyped under the presumption that they were *F. pennsylvanica* and explain that the 20 cultivars were from a *Fraxinus* cultivar collection. We included the passport data for all cultivars in the original submission. In the revised introduction we now state “In this investigation we genotyped 48 naturally regenerated populations of *F. pennsylvanica* (1291 trees) and 20 cultivars with the same set of 16 EST-SSR markers to detect first-generation progeny from cultivars in the populations we genotyped and assess the impact of such gene flow on population differentiation and population substructure”.

6. Hypothesis have been discussed in the discussion but clear Hypothesis were not given in the introduction section.

We have addressed this in the revisions to the abstract and introduction. In the revised abstract, in the second sentence we now write “. Undetected cultivar gene flow, if extensive, could significantly lower genetic diversity within populations, suppress differentiation between populations, generate interspecific admixture not driven by long-standing natural processes, and affect the impact of abiotic and biotic threats. In this investigation we generated the first range-wide genetic assessment of *F. pennsylvanica* and included genotypes from 20 commercial cultivars to detect the extent of cultivar gene flow into natural stands.”

7. What is the role of emerald ash borer in depleting the population of Green or White ash? or Cultivar proven to be better resistant form emerald ash borer not discussed.

Population depletion due to the emerald ash borer and possible resistance to the emerald ash borer in the cultivars are outside the scope of this investigation, although we do address the importance of these considerations in the discussion. In the methods, we indicate that we avoided those areas in which EAB has inflicted the most mortality: “We did not collect samples in southern Michigan, northern Indiana, or northern Ohio, as surviving adult trees were unlikely to be representative of the genetic diversity existing before the emerald ash borer invasion”. While the data we have does not address the questions raised by reviewer, the genetic impact of EAB-inflicted mortality and the evaluation of cultivars for response to EAB infestation are important topics for future investigations.

8. Over all impression of the manuscript is highly confusing with no clear cut objectives and what they achieve by doing the whole study? Do cultivar resulted in depletion of allele richness on Green ash or white ash population? Which species needed to be conserved?

We hope that the revisions referenced above clear away the confusion over species distinctions. The methods and the figure provided clearly show that the association between allele richness and cultivar gene flow arises from a linear regression that includes the data from all of the populations, regardless of interspecific admixture. In the discussion we argue that “Conservation of the gene pools of F. pennsylvanica, F. americana and the other North American Fraxinus under threat from EAB requires consideration of what “gene pool” means given the evidence for locally extensive gene flow from cultivars and porous species boundaries.” The data we have supports the expectation of low species boundaries, at least under some circumstances, but this is a matter for separate investigation.
